# An Embedded System Using Convolutional Neural Network Model for Online and Real-Time ECG Signal Classification and Prediction

**DOI:** 10.3390/diagnostics12040795

**Published:** 2022-03-22

**Authors:** Wahyu Caesarendra, Taufiq Aiman Hishamuddin, Daphne Teck Ching Lai, Asmah Husaini, Lisa Nurhasanah, Adam Glowacz, Gusti Ahmad Fanshuri Alfarisy

**Affiliations:** 1 Faculty of Integrated Technologies, Universiti Brunei Darussalam, Jalan Tungku Link, Gadong BE1410, Brunei; taufiqaiman.hisham@gmail.com; 2 Institute of Applied Data Analytics, Universiti Brunei Darussalam, Jalan Tungku Link, Gadong BE1410, Brunei; daphne.lai@ubd.edu.bn; 3 Institute of Health Sciences, Universiti Brunei Darussalam, Jalan Tungku Link, Gadong BE1410, Brunei; asmah.husaini@ubd.edu.bn; 4 Physical Medicine and Rehabilitation Department, Faculty of Medicine, Diponegoro University, Semarang 50275, Indonesia; lisanurhasanah.dr@gmail.com; 5 Department of Automatic Control and Robotics, Faculty of Electrical Engineering, Automatics, Computer Science and Biomedical Engineering, AGH University of Science and Technology, 30-059 Kraków, Poland; adglow@agh.edu.pl; 6 Department of Informatics, Kalimantan Institute of Technology, Jl. Soekarno Hatta KM. 15, Balikpapan 76127, Indonesia; gusti.alfarisy@lecturer.itk.ac.id

**Keywords:** convolutional neural network (CNN), deep learning, ECG images classification, ECG online prediction

## Abstract

This paper presents an automatic ECG signal classification system that applied the Deep Learning (DL) model to classify four types of ECG signals. In the first part of our work, we present the model development. Four different classes of ECG signals from the PhysioNet open-source database were selected and used. This preliminary study used a Deep Learning (DL) technique namely Convolutional Neural Network (CNN) to classify and predict the ECG signals from four different classes: normal, sudden death, arrhythmia, and supraventricular arrhythmia. The classification and prediction process includes pulse extraction, image reshaping, training dataset, and testing process. In general, the training accuracy achieved up to 95% after 100 epochs. However, the prediction of each ECG single type shows a differentiation. Among the four classes, the results show that the predictions for sudden death ECG waveforms are the highest, i.e., 80 out of 80 samples are correct (100% accuracy). In contrast, the lowest is the prediction for normal sinus ECG waveforms, i.e., 74 out of 80 samples are correct (92.5% accuracy). This is due to the image features of normal sinus ECG waveforms being almost similar to the image features of supraventricular arrhythmia ECG waveforms. However, the model has been tuned to achieve an optimal prediction. In the second part, we presented the hardware implementation with the predictive model embedded in an NVIDIA Jetson Nanoprocessor for the online and real-time classification of ECG waveforms.

## 1. Introduction

Coronary heart disease is one of the most common causes of death today. Coronary heart disease is considered the largest contributor to death in the United States with a percentage of 18%. This percentage is expected to increase by as much as 100% by 2030 [1]. In addition to the United States, coronary heart disease sufferers are also high in South Asian countries. Some of the coronary heart disease sufferers in South Asia are still at an early age [2]. Several causes of coronary heart disease, including unhealthy food, stress level, dietary habits, minimal exercise, smoking, and consuming alcohol. In addition, other health problems such as cholesterol, high blood pressure, and diabetes can also cause coronary heart disease [1].

Coronary heart disease detection is done by observing the morphology of the electrocardiogram (ECG) signal. This signal contains the electrical activity that occurs in the human heart. If there is a disturbance in the heart, an abnormality in the ECG signal will be identified [3]. The ECG signal consists of several types of waves, including P waves, QRS waves, and T waves. These waveforms will be analyzed to determine the presence of cardiac disorders [4]. The most important information in the ECG waveform is found in the morphology of the P wave, QRS complex, and T wave.

The ECG waveform remains an essential part of diagnosis and treatment for human cardiac in decades. However, diagnosing of the ECG waveform is challenging due to the complexity of ECG signal morphology in nature. The process of analyzing the morphology of the ECG signal for the signal classification process must be carried out by an expert [5]. To become an expert, it takes years of study to be able to analyze the morphology of ECG signal correctly [6]. The results of manual analysis by this expert are sometimes inaccurate due to human error caused by fatigue. There is a large number of ECG waveform parameters that need to be measured in the ECG waveforms [5]. Therefore, it is necessary to analyze and classify the ECG signal automatically using a computer.

By using a computer, the classification results are expected to be more accurate. In addition, the classification process can be carried out faster than the classification process by cardiologists [6,7]. There are many studies using hardware implementation with the aim of helping experts in detecting heart abnormalities quickly. Gawali et al. [8] develop a real-time ECG sensor that can acquire and store patient ECG data in real-time. Such patient data can be used for any real-time processing application. Jeon et al. [9] developed a personal, real-time portable ECG device using a digital filter-based hardware architecture. This development aims to help patients monitor their heart condition daily, at a low cost. Sadhukhan et al. [10] proposed the identification of myocardial infarction using the distribution pattern in the harmonic phase of the ECG data, which have a simple computational feature so that the identification process becomes faster and easier.

To perform the ECG classification process, hardware requires a system that can detect and classify the input ECG signal correctly. Therefore, an appropriate architecture is needed to classify or detect cardiac abnormalities correctly. Classification of computer-based automatic ECG signals can be done using the Machine Learning (ML) method. Celine et al. [11] used several machine learning methods such as Artificial Neural Network (ANN), Naive Bayes (NB), Support Vector Machine (SVM), and Adaboost classifier. The accuracy obtained from these methods ranges from 87% to 99%. Machine Learning is one part of Artificial Intelligence (AI) that allows computers to understand patterns so they can make decisions from existing data sets [12]. A number of ML methods have been progressively developed over the past few decades for wide number of applications [13,14]. The ML method is also applied for predictive analysis of heart rates as presented in [15]. Learning models in machine learning are divided into three types: supervised learning, unsupervised learning, and reinforcement learning [16]. However, machine learning cannot extract features automatically. The features learned in machine learning are features extracted manually. This makes the learning outcomes of the model still very dependent on humans. In addition, the features obtained are only superficial features and it takes longer to build a successful activity recognition system [17].

To overcome this, the Deep Learning (DL) method was introduced. DL appears to excel in a relatively short time to solve many complex problems. It has a better ability to display representations at an abstract level than general ML methods. Its architecture is capable of extracting hierarchical representations of data automatically and then utilizing the rest of the stacked layers to learn complex features from simpler ones. Thus, DL does not require further human intervention in the feature extraction process [18]. In addition, DL has proven to be better than other ML techniques, such as SVM, because DL can use multiple layers and transformations, compared with the two layers used by SVM [16]. DL applications have also been developed both for small-scale to large-scale data [18,19]. One of the superior performances of DL methods is the image and video processing capability and this capability has been applied widely for contactless heart rate measurement [20]. A study reported the application of DL for heart rate modeling and prediction is presented in [21].

In conventional ML methods, the features are always extracted via a heuristic and handcrafted way. It relies on human experience or knowledge. Human knowledge may help in a certain task-specific setting. However, in more general environments and tasks, it is different and can result in a lower chance of and taking a longer time to build a successful activity recognition system. In addition, only shallow features can be learned according to human expertise [17]. In previous work [19], the drawback of conventional ML compared with DL for ECG classification was presented. The morphology of ECG signals shows significant variation in different patients under different physical conditions. DL tends to overcome those limitations by calculating the learning features automatically without conventional handcrafted features. Deep learning can be trained on large-scale labeled datasets. Wang et al. [22] surveyed sensor-based human activity recognition using a deep learning algorithm. Three new advances are highlighted: sensor modalities, deep models, and applications.

Recent technologies developed during the COVID-19 pandemic have created a new perspective on the application of AI in telemedicine platform monitoring of heart diagnosis [23,24,25]. A comprehensive review of the recent advances of DL-based methods for remote heart rate measurement is presented in [23]. The selected methods in the review paper were categorized based on the model architecture and application. An automated patient monitoring in homecare assistance for heart predictive diagnosis is presented in [24]. The automated smart health system used multilayer perceptron (MLP) ANN as a predictive method. The system also consists of control room, homecare smart sensors, and could for big data platform. A decision support system (DSS) based on SVM and Long Short-Term Memory (LSTM) for predicting the health status is presented in [25]. The DSS is composed of different sensors, and one of them is ECG sensor.

This research investigates the performance of a common DL method namely Convolutional Neural Network (CNN) in ECG signal classification. The CNN is a feed-forward network, which comprises convolution, pooling, and fully-connected layers [26]. The operation acts as a feature extractor by learning from the diverse input signals in a series of convolutional layers. The extracted features can be used for classification in subsequent layers. The pooling operation is employed to reduce the spatial dimension of the input sample while retaining significant information. With fully-connected nodes, a weighted sum of all the outputs from the previous layer is established to determine a specific target output. The segmentation of ECG into the 1-dimensional CNN for training and testing in classification. Conceptually, the CNN method can reduce the burden during training as a good feature extraction technique for the automated detection of ECG signal. CNN can eliminate the need for preprocessing and separate feature extraction technique [27]. In other words, the feature extraction, feature selection, and classification stages are merged in the CNN algorithm.

Then, the CNN model that has been built will be implemented on the hardware to classify the ECG signal data to be given. The way it works is that the input in the form of an ECG signal image will be captured through the camera and will be classified using the CNN algorithm built and embedded in the hardware. The CNN model will predict the type of ECG signal that is entered, and the prediction results will be displayed on the monitor screen. This is very useful for classifying ECG signals more quickly and easily.

## 2. ECG Image Data and Preprocessing Method

**Figure 1:**
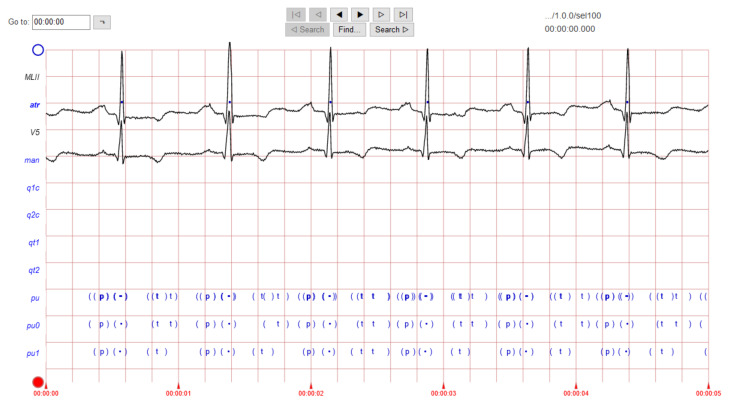
ECG image data for ‘normal sinus’ waveform.

**Figure 2:**
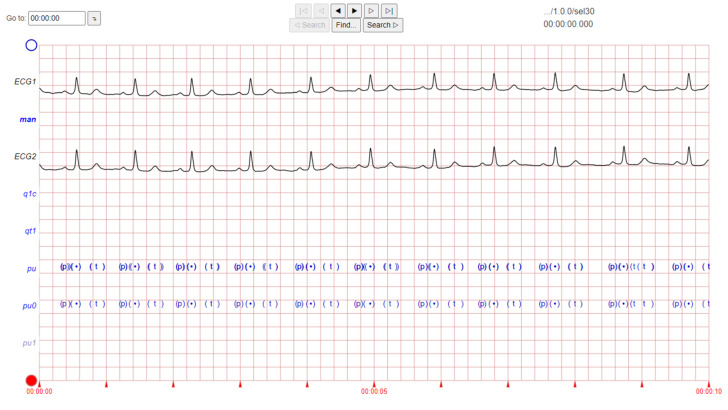
ECG image data for ‘sudden death’ abnormality.

**Figure 3:**
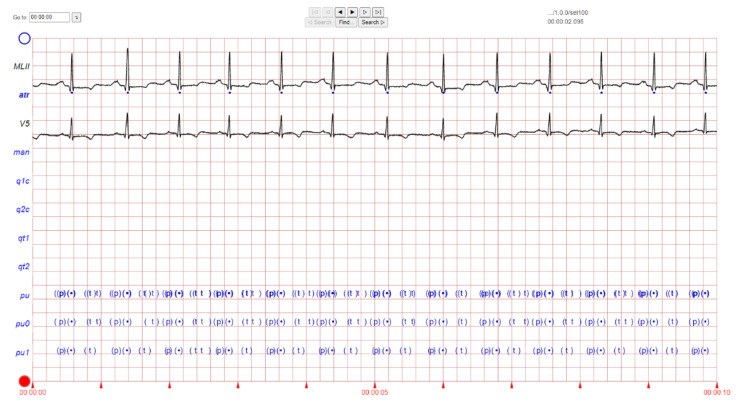
ECG image data for ‘arrhythmia’ abnormality.

**Figure 4:**
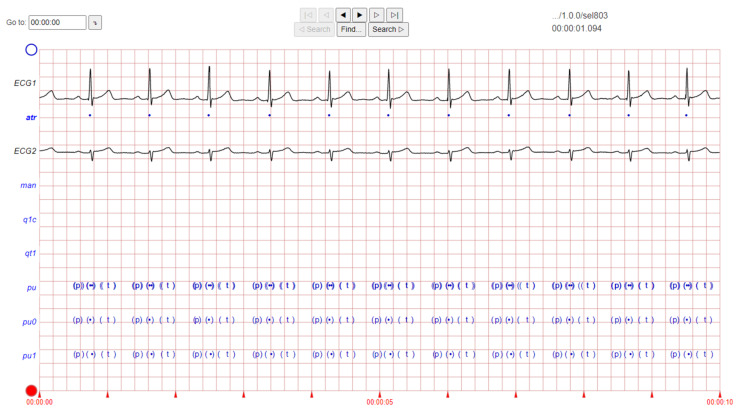
ECG image data for ‘supraventricular arrhythmia’ abnormality.

The ECG images that were used as data input for the CNN were obtained from an open-source database, namely, the QT Database from the PhysioNet [28]. An example of ECG images, i.e., ‘normal’, ‘sudden death’, ‘arrhythmia’, and ‘supraventricular arrhythmia’, waveform is presented in Figure 1, Figure 2, Figure 3 and Figure 4. These data were preprocessed prior to building a CNN model for ECG classification and prediction. Four classifications have been selected from several patients, with multiple images taken from each patient. Images were manually cropped from one recording signal that consist of one complete pulse (P wave, QRS complex, and T wave), with the exceptions of cardiac abnormality categories, where certain visual features may be absent. The objective of the cropping is to simulate the camera capture during the development of the online and real-time ECG signal classification and prediction presented in Section 5.

**Figure 5:**
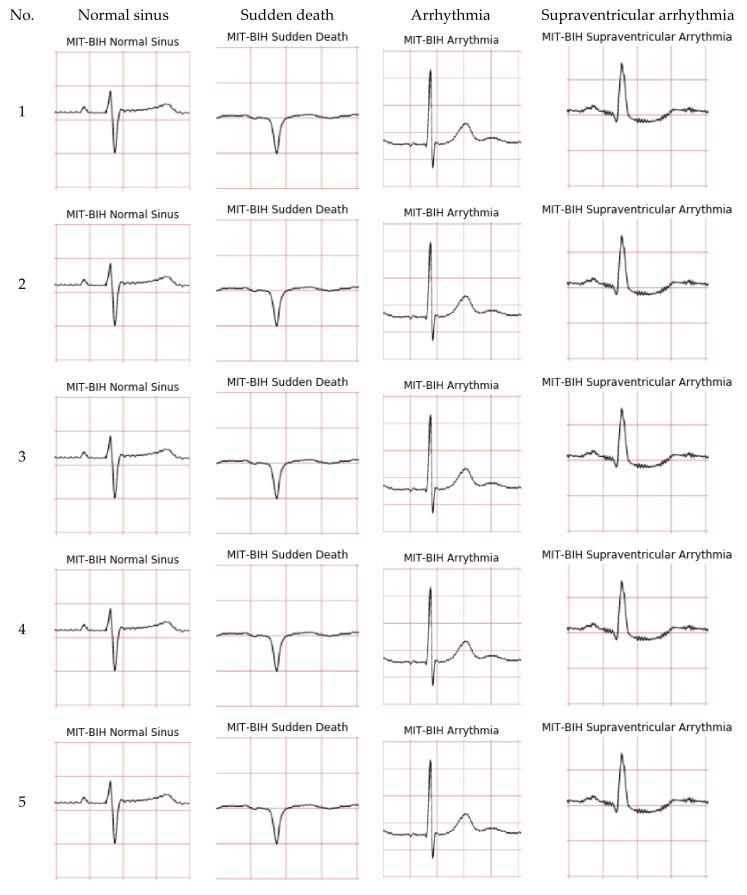
Preprocessed of ECG images for CNN data input.

An example of data/image preprocessed with a uniform length and width is presented in Figure 5. Figure 5 includes the preprocessed images for ‘normal sinus’, ‘sudden death’, ‘arrhythmia’, and ‘supraventricular arrhythmia’ ECG waveform which are originally presented in Figure 1, Figure 2, Figure 3 and Figure 4, respectively.

## 3. Convolutional Neural Network (CNN) Method and Data Processing Stage

A convolutional neural network (CNN) is a type of deep learning algorithm that consists of various functions such as local perceptions of images, sharing weights, filters, subsampling, fully connection, and multi-classification. In the classification and prediction of multi-classes of ECG waveforms, the first step of CNN is to generate local perceptions of images. CNN is a deep learning method that is often used in hardware implementations for image classification [27].

There are three types of layers of CNN, namely convolution layer, pooling layer, and fully connected layer. The input data feature extraction process is carried out at the convolution layer. This feature extraction process is carried out by several filters. The size of the height and weight of the filter is less than the input that goes into the convolution layer. Each filter and input will be connected to form a series of neurons. The pooling layer is a layer after the convolution layer. This layer reduces the dimensions of the features and the number of parameters of the processing. The fully-connected layer is the last layer of CNN. This layer is in charge of classifying according to the number of existing classes. This classification process is carried out by considering the features that have been obtained from the previous process [28].

**Figure 6:**
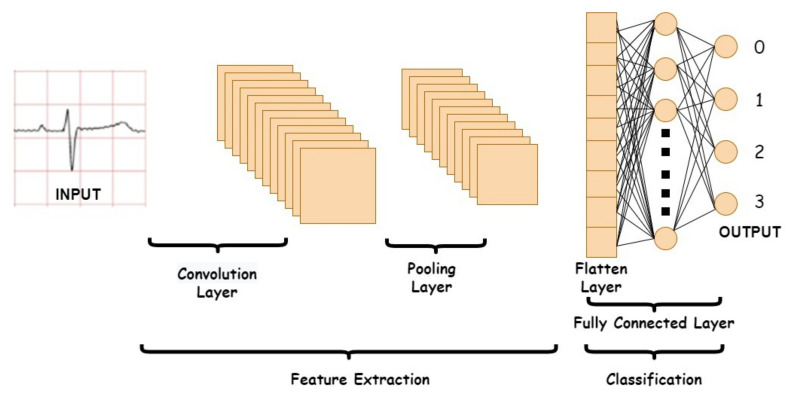
CNN architecture.

The convolution layer is the key layer in CNN. This layer will extract each input matrix and will generate a new output matrix. The output matrix contains important features that will be useful for the classification process. The process at the convolution layer is shown in Figure 6. The equation to calculate the image filter for each pixel in the convolution layer is [29]

(1) $${A_{j}}_{\;}=f\;(\sum_{i=1}^{N}I_{i}*K_{i,j\;}+B_{j})$$

*A_j_* is the output of the matrix on the convolution layer, f is a non-linear activation function, *I_i_* is the input matrix in the *K_i,j_* kernel, and *B_j_* is the bias value that will be added to each matrix element.

The pooling layer has a function to reduce dimensions. The matrix from the convolution layer output will be reduced in dimension to speed up the computation process [30]. Several ways are commonly done, namely, by using max-pooling, minimum-pooling, or average-pooling [31]. The 2D matrix from the pooling layer will be converted into a 1D matrix on the Flatten layer. In this layer, all matrix elements will be placed in a 1D array before entering the fully connected layer. At the fully connected layer, the classification process will be carried out [31]. The architecture of CNN is presented in Figure 6.

In this study, datasets of four classes of images, i.e., ‘normal’, ‘sudden death’, ‘arrhythmia’, and ‘supraventricular arrhythmia’, are collected to provide a sample image as a local perception of an image. Different convolutional areas are usually used to calculate weights in order to reduce the calculation time. In the filters step, the CNN builds many filters which extract various kinds of features. In subsampling, the CNN employs subsampling (also called downsampling) to extract features that are invariant to translation, hence reducing the calculation complexity. This state has differed from traditional machine learning methods that requires handcrafted features for training which may be time-consuming and prone to error. When fully connected, the CNN connects all extracted features through many convolutions and pooling.

**Figure 7:**
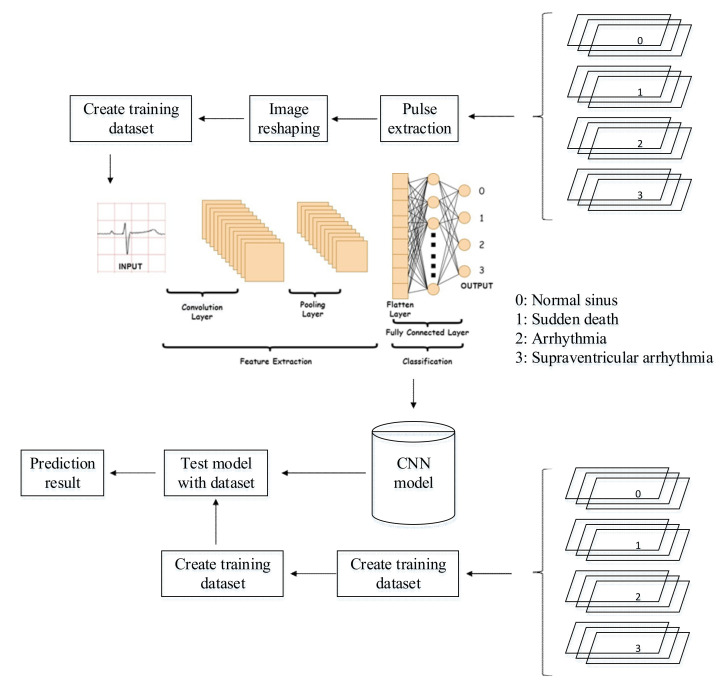
CNN method flowchart.

Once all the images have been extracted, they are loaded into the CNN model. The images are adjusted to a uniform length and width for consistency. They are then compiled into a training dataset, with a validation split, where a certain percentage of the image samples will be used for validation. The preprocessed ECG images are stored into cached memory to improve the efficiency of learning iteration speed for the CNN model. The methodology for this work is illustrated in Figure 7. It can be seen in the flowchart that the training data required a set of data set that consist of four classes. Once the pre-processing part is completed, the preprocessed data are fed into the CNN architecture to build a classification and prediction model. The CNN model is then tested with an individual ECG waveform as presented in Figure 7.

**Figure 8:**
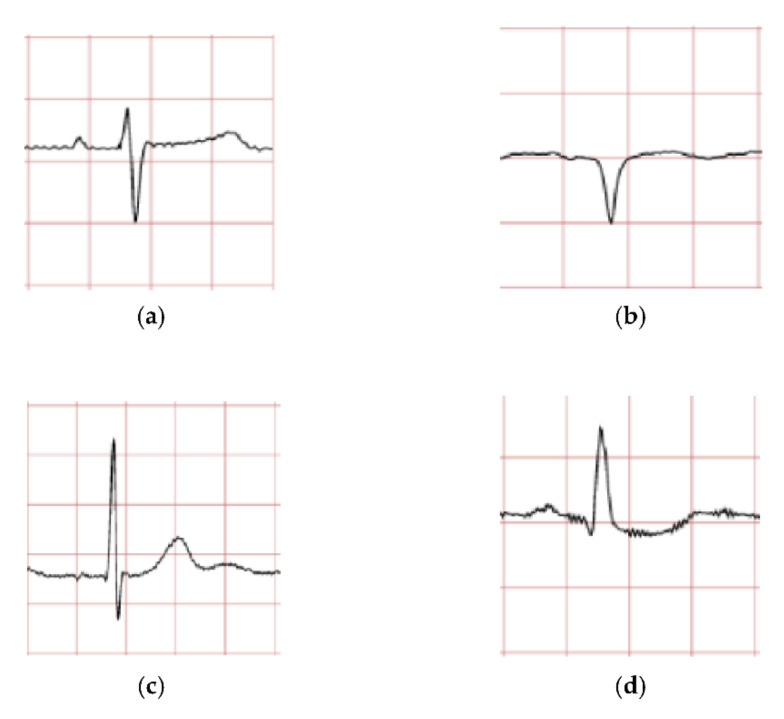
ECG data preprocessed: (**a**) normal sinus ECG waveform; (**b**) sudden death ECG waveform; (**c**) arrhythmia ECG waveform; (**d**) supraventricular arrhythmia ECG waveform.

The model consists of multiple layers, each with a defined number of filters, with certain types of layers requiring padding and activation types. The preprocessed images presented in Figure 8 are stored in digital numerical form, and the rescaling layer allows image values from 0 to 255 to be set between 0 and 1. This is to normalize the image data. Convolution and pooling layers are responsible for the extraction of features for each image. Once the features have been extracted, the image data needs to be flattened from a 2D array into a 1D array, before proceeding through dense layers in which the CNN learns via identifying features from image data and matching them to their correct labels.

Aiming to prevent overfitting of the model, transfer learning as a feature extractor is employed. VGG16 is utilized with ImageNet pre-trained weights which consist of 13 convolutions and three fully-connected layers [32]. We choose VGG16 due to its simplicity and popularity in achieving good results by extending more layers.

In the classification part of the model, we replace the last 3 layers with our proposed architectures as presented in Table 1. Originally, two layers before output contains roughly 119 million parameters using two consecutive fully-connected layers with 4096 neurons. We propose to reduce the connection by using 256 neurons only which reduces the number of parameters significantly. Decreasing the neurons is intended to avoid unnecessary parameters to be trained. Our problem also uses smaller number of classes that reduce the need of parameters.

We add LeakyReLU as a non-linear activation function, as it may increase speed training and break the zero slope of the negative input from the standard ReLU. Empirical study also shows that this nonlinear function applied at the fully-connection part outperforms the standard ReLU in terms of transfer learning [33]. Furthermore, dropout layers are also added to help attain the generalization of our model [34]. It is a strong generalization technique with an inexpensive cost that deactivates some units during training. Dropout can be seen as ensemble of subnetworks. The probability of units being deactivated is denoted as *p*.

## 4. Results and Discussion

### 4.1. Hyperparameter Settings

The hyperparameters and configuration of the CNN model are described in Table 2. A learning rate of 1 × 10^−5^ Adam was employed as the optimizer due to its popularity and capability in adaptive learning rate. The image resolution was fixed to 256 × 256. Meanwhile, cross entropy loss was used as the standard loss for classification task in deep learning. The proposed model was implemented using Tensorflow.

The dataset was divided into training and validation dataset. We used the validation dataset to validate the performance of trained model. Meanwhile, epochs are the number of times that the CNN will repeat the process of feeding all the image data in training dataset into the CNN layers. We set epoch to 100 and depicted the accuracy and loss score to observe the overfitting occurrence. Mini-batch size dictate the sample of trained dataset that is used to derive gradient and update the parameters of the CNN.

### 4.2. Training and Validation Performance

The CNN model is trained to classify four ECG waveforms: ‘normal’, ‘sudden death’, ‘arrhythmia’, and ‘supraventricular arrhythmia’. The ‘normal’ ECG images show clear common features at normal amplitudes, such as P wave, QRS complex, and T wave. ‘Sudden death’ display lack of amplitude change and may have missing features. ‘Arrhythmia’ can be seen to have ‘jitter’ and unclear features, whereas ‘supraventricular arrhythmia’ is similar to the former but shows larger fluctuations and variations in ECG patterns from the norm.

**Figure 9:**
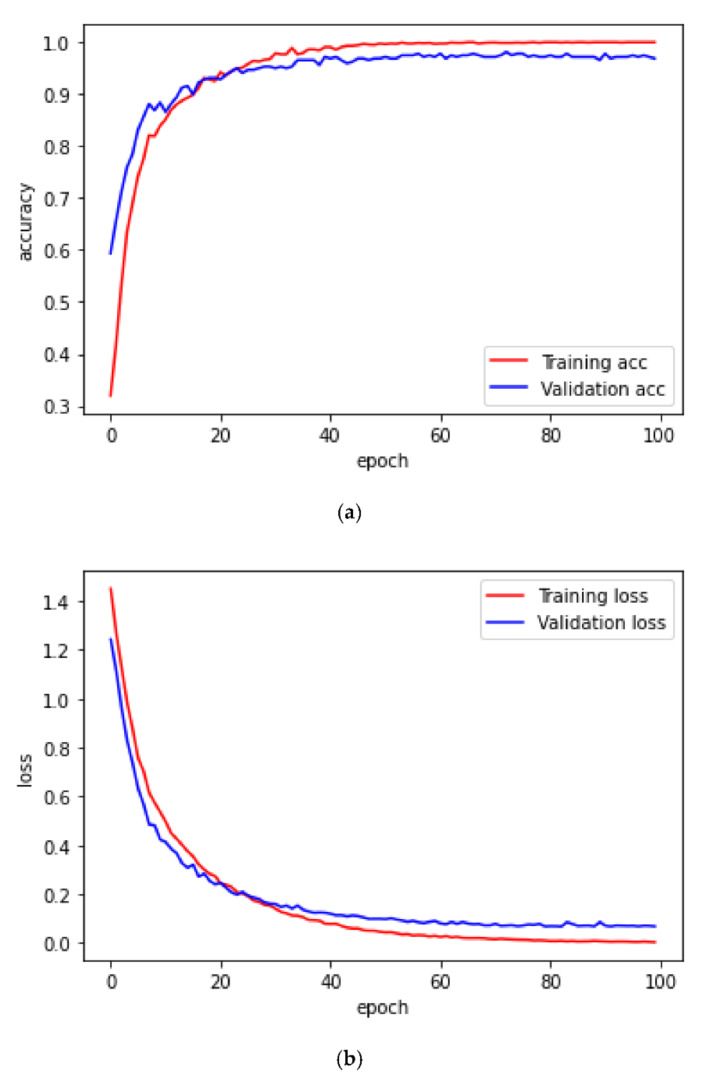
(**a**) Training/validation accuracy and (**b**) loss trend with each epoch.

Figure 9 shows the comparison between training and validation accuracy and loss per epoch. As can be seen from the accuracy plot, the training and validation accuracy maintains steady increments before stabilizing within a range of values. However, in the loss graph, the two loss plots lead towards zero, but after a certain number of epochs, the validation loss diverges from the training loss.

### 4.3. CNN Prediction

In this study, 80% datasets (1280 ECG images) were used for training and the remaining 20% datasets (80 ECG images) were used for testing. The 1280 ECG training images consist of 80 ECG mages for each class. A CNN prediction of testing dataset is presented in Figure 8. The testing datasets used were 20 ECG images for each class and 80 ECG images in total (four classes).

**Figure 10:**
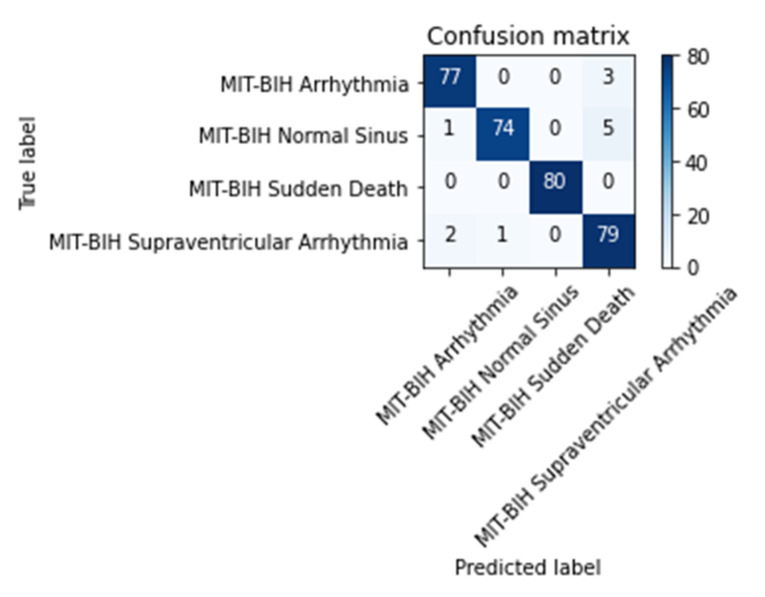
Confusion matrix of CNN prediction of testing data (80 ECG images for each class).

According to Figure 10, 80 images of each class were tested for classification and prediction. For ECG ‘arrhythmia’ class, 77 images were classified or predicted correctly, however, three images were misclassified to the ‘supraventricular arrhythmia’ class. In ECG ‘normal sinus’ prediction, 74 images were classified in a proper class; however, there was one image predicted as ‘arrhythmia’ and five images were predicted as ECG ‘supraventricular arrhythmia’ class. An outperformed result compared to others was achieved in ECG ‘sudden death’ class with 100% correct classification result. In the last class, i.e., ECG ‘supraventricular arrhythmia’, 79 images were predicted correctly and two images were misclassified to ECG ‘arrhythmia’ and one image predicted as ECG ‘normal sinus’ class.

**Figure 11:**
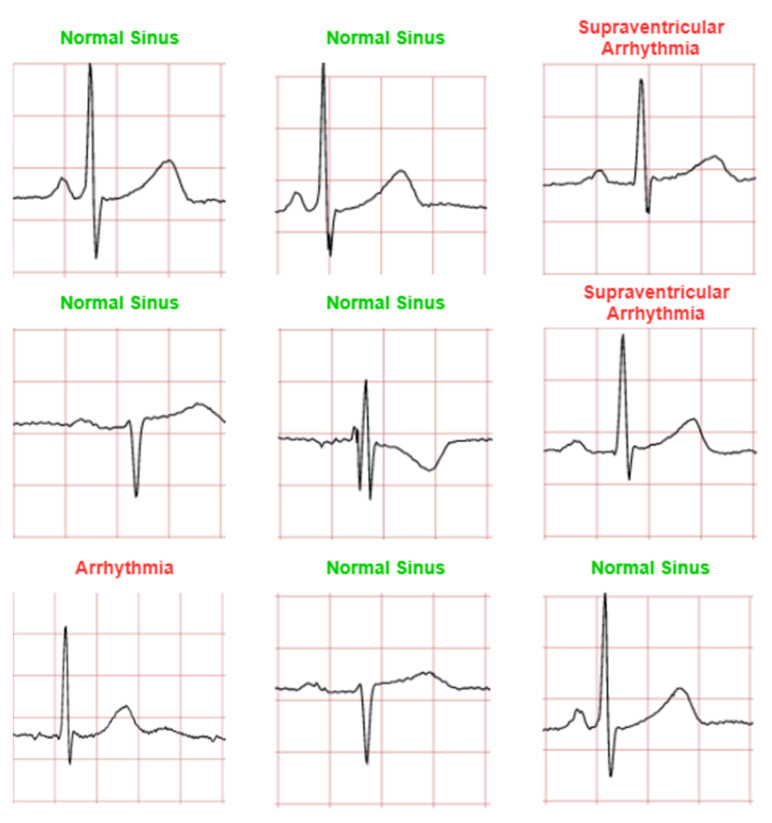
Prediction for ‘normal sinus’ ECG waveforms. (Note: red and green title indicated incorrect and correct prediction, respectively).

**Figure 12:**
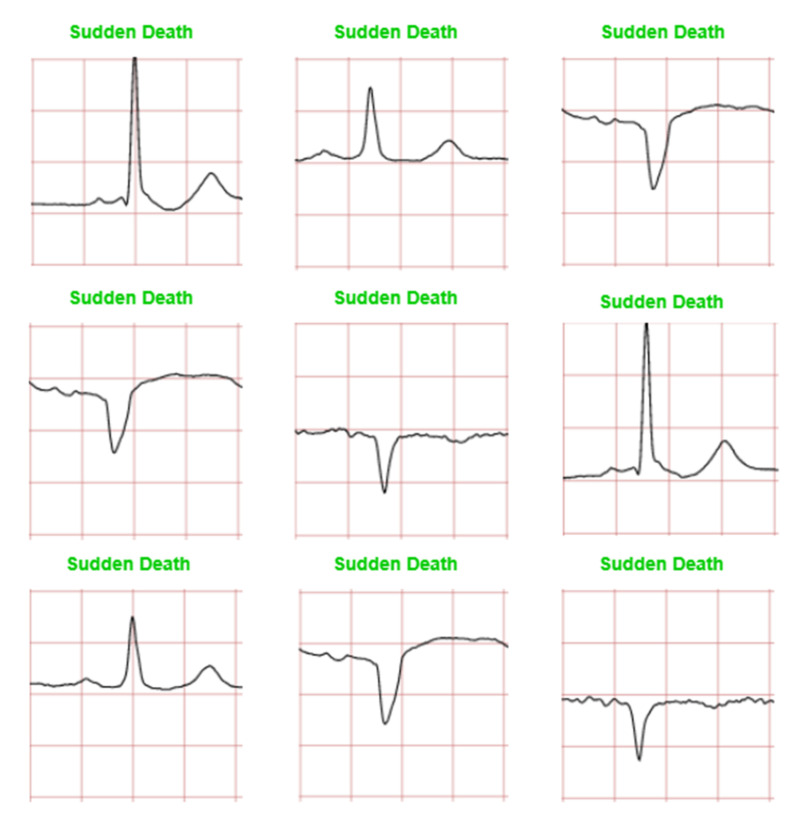
Prediction for ‘sudden death’ ECG waveforms. (Note: red and green title indicated incorrect and correct prediction, respectively).

**Figure 13:**
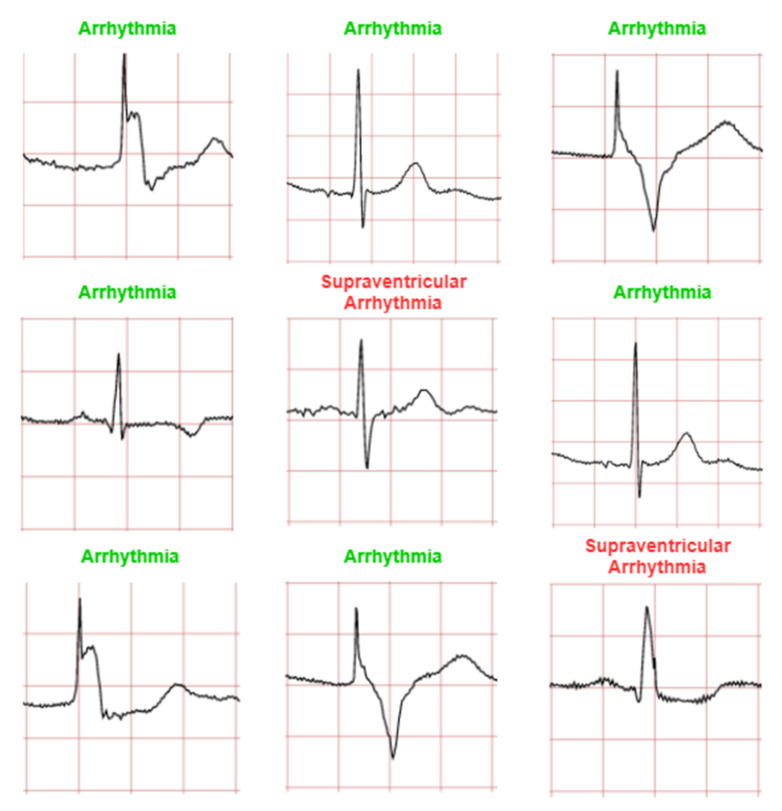
Prediction for ‘arrhythmia’ ECG waveforms. (Note: red and green title indicated incorrect and correct prediction, respectively).

**Figure 14:**
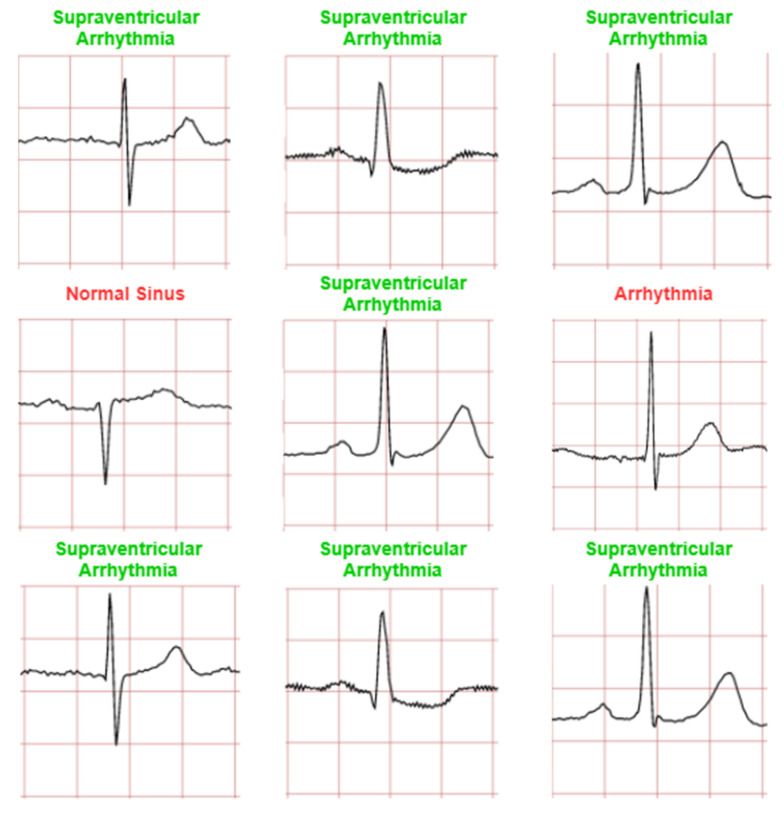
Prediction for ‘supraventricular arrhythmia’ ECG waveforms. (Note: red and green title indicated incorrect and correct prediction, respectively).

Figure 11, Figure 12, Figure 13 and Figure 14 show the CNN model predictions with lower datasets, i.e., only 10 images for visualization purpose prior to the hardware implementation. For ‘normal sinus’ ECG images testing shows in Figure 11, one ECG image was miss predicted to ‘arrhythmia’ and two ECG images were classified as ‘supraventricular arrhythmia’. A consistent prediction result as 80 testing images was presented in Figure 12 for sudden death’ prediction where a 100% ECG images were predicted correctly.

It can be observed that among the four classifications, ‘normal sinus’ can be seen as a lowest prediction with 70% accuracy (for 10 ECG images testing case). Some of the incorrect predictions in the other classifications include this anomalous classification, and if not once, being the dominant erroneous prediction. One argument that can be drawn to justify this may be the irregularity in the waveform shape of normal sinus ECG. While other classifications have notable features especially in sudden death with achieved a 100% prediction accuracy for 10 images testing case. ‘Arrhythmia’ and ‘supraventricular arrhythmia’ also have irregular features, however a CNN can still detect the different with result in 80% accuracy for 10 ECG images testing case. In general, this irregular classification affects the other classifications, as it mimics their features.

A detail of the training and validation result is presented in Table 3. One-hundred epochs are used for the training and validation. It can be seen that the training and validation accuracy decrease as the number of epochs decrease.

## 5. Development of Online and Real-Time ECG Image Classification and Prediction

**Figure 15:**
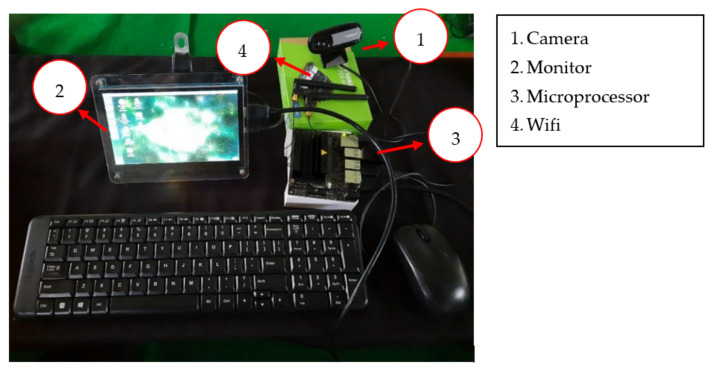
Equipment used for ECG image detection in hardware implementation.

The CNN model that has been built was implemented on a hardware device to classify the new ECG signal. The prototype of the hardware implementation that was built can be shown in Figure 15. The hardware devices used include cameras, embedded systems (NVIDIA^®^ Jetson Nano), and monitor. The camera is used to capture images of the ECG signal to be classified with general specifications: field of view (FOV) 60°, focal length 4.0 mm, optical resolution (true) 1280 × 960 1.2 MP, and frame rate (max) 30fps @ 640 × 480. The microprocessor used was a NVIDIA^®^ Jetson Nano with general specification as follows: GPU 128-core NVIDIA Maxwell™, CPU Quad-core ARM^®^ A57 @ 1.43 GHz, Memory 4, GB 64-bit LPDDR4 25.6 GB/s.

**Figure 16:**
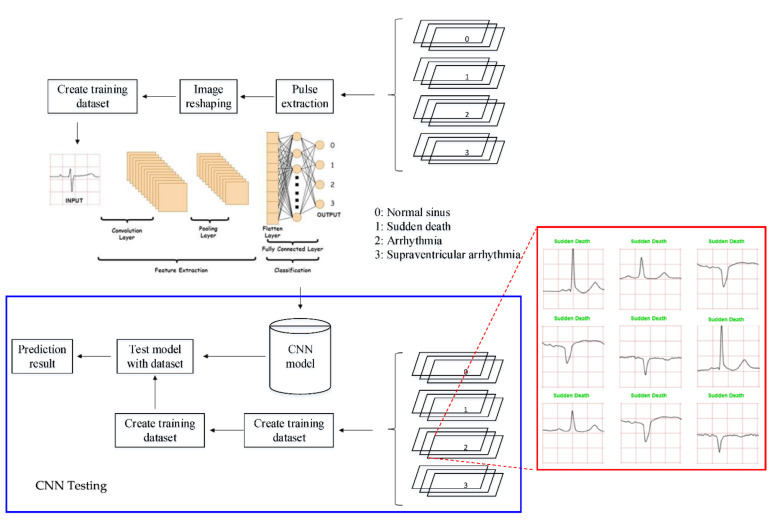
Prediction for ‘supraventricular arrhythmia’ ECG waveforms. (Note: red and green title indicated incorrect and correct prediction).

**Figure 17:**
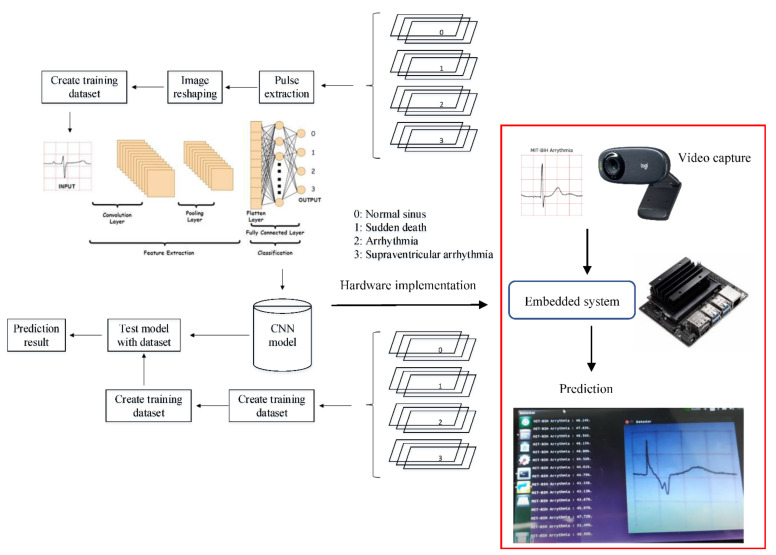
Online and real-time ECG image prediction in hardware implementation.

An illustration of the connection between the CNN model and the prediction of one case is presented in Figure 16. This illustration demonstrates the implemented idea of the CNN prediction model in the hardware as a preliminary study for online ECG image detection. A hardware implementation is presented in Figure 16 and Figure 17. The images captured by the camera are processed and classified using the CNN model which has been implemented into the embedded system (NVIDIA^®^ Jetson Nano). The results of the image classification are displayed on the monitor.

There are two main processes in building a deep learning model: the training process and the testing process. The dataset used will be divided into two for training purposes and testing purposes. The dataset used for training must be more than the testing dataset. The dataset which is divided into four classes—normal, sudden death, arrhythmia, and supraventricular arrhythmia—will be processed using pulse extraction which will extract the image dataset into binary codes so that it can be processed in the computational process. Then, the dataset will be manipulated to optimize the training process with image reshaping. After that, the ready dataset will go into the cache memory for the implementation of the CNN algorithm. The testing process is the same as the training process. The processed testing dataset is used to evaluate machine learning models. If the model performance is optimal, then the model can be implemented on hardware.

In this study, the CNN model that has been built will be embedded into the system on the NVIDIA^®^ Jetson Nano. Later, this CNN model will classify the input ECG signal image. The input image will be taken through the camera. The predictive result of the image input will be displayed on the monitor.

A video result for online prediction and detection of one class ECG waveform (image) based on the CNN method and NVIDIA^®^ Jetson Nano processor is presented in the following YouTube link in Supplementary Materials.

## 6. Limitations

This work has focused on developing and deploying a classification system to analyze ECG waveforms and detect heart diseases in real-time. This system has demonstrated good accuracy as a real-time decision support tool. However, one factor that affects accuracy not considered in this paper is a standard protocol for image capture such as distance between image and camera and preprocessing such as rescaling, adjusting image orientation, etc. prior to model training. This affects the consistency of images such as image scale, brightness, quality, and, in turn, the future image collection and analysis processes. Thus, further investigation to improve model robustness and prevent overfitting through analysis of other volumes of ECG waveforms from different formats and sources is needed. This would also involve investigation of data augmentation techniques using ImageDataGenerator from Keras Python Library and other approach with the model towards establishing an effective and precise standard protocol of image capture and processing.

## 7. Future Work

According to World Health Organization (WHO), heart disease is still a leading cause of death in the world, especially in Southeast Asia [35]. As technology advances, it is expected that heart disease can be detected as early as possible. The supporting examination device that is easily available today is electrocardiography (ECG) but reading the results of the examination still requires interpretation from an expert or a more complete ECG. In addition, there are not many cardiologists available in hospital and clinic especially in suburban area or remote are and far away from capital or big cities compare to a number of heart disease patients.

Therefore, in this study, a device that is easy to use and more affordable is developed to help interpret the results of a simple ECG examination that can be carried out by any health worker. The authors wishes that this instrument can be utilized as a clinical reference. However, this instrument still needs further clinical examination and trials by comparing the reading of this instrument to the gold standard examination, i.e., a standardized ECG or reading by cardiologists and specialists.

As previously highlighted, one limitation of our work, and also out of current scope of this work, is the standardized protocol. This is particularly important for the model development from images that may be inconsistently collected. Such inconsistency can be in the scale of the taken photo, the resolution, file format, lighting conditions, camera and ECG printout distance, and so forth. We hope to address this in our future work with two approaches to consider, standard hardware setup, and/or software manipulations.

**Figure 18:**
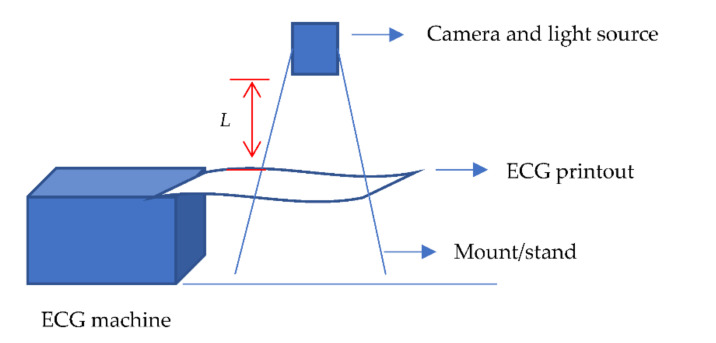
Illustration of possible hardware setup for investigating standard image capture with predetermined *L* distance from the ECG printout to the camera.

For the hardware setup investigations, a standard mount for the placing of the camera lens will be investigated. Other considerations include the height of the mount, and therefore the distance between camera lens and ECG printer, different camera types and make, light source and mount position on top of the printer, as illustrated in Figure 18.

For software treatments of taken photographs, we hope to maintain consistency and evaluate the precision of the proposed system through investigation in image processing and correction protocol including data augmentation [36], image reconstruction [37,38,39] and image segmentation [39,40], and model training methods such that consistent images can be produced, that can robustly handle or remove such inconsistencies in the images collected. The consistency of processed images and effect on model accuracy will be measured, studying the effects from camera and light source mount settings.

For hyperparameter settings, it requires human experts to determine beneficial choices. The settings may lead to non-optimal options using manual tuning especially targeted for embedded hardware. Hyperparameter optimization such as Bayesian Optimization [41] can be utilized for searching optimal architecture and other hyperparameters by accounting for the latency and the number of parameters. In addition, considering a deep model specifically designed for mobile devices such as MobileNetV3 [42] will be considered. By combining the MobileNetV3 and the hyperparameter optimization, we hope that a robust model with smaller latency, a smaller number of parameters, and higher accuracy could be achieved.

## 8. Conclusions

Heart disease is one of the most common causes of death. Therefore, a rapid diagnosis process is needed to reduce the risk of death from heart disease. The process of diagnosing heart disease is done using ECG signals. Currently, the automatic diagnosis of heart disease using deep learning methods is very popular. A method for automated classification and prediction of multi classes ECG waveforms based on the convolutional neural network (CNN) method is presented. The embedded CNN method is capable to classify and predict the four different ECG waveforms with a real-time camera. The proposed CNN model implemented on hardware is potential to assist doctors in diagnosing heart disease.

According to the discussion with a few of specialists and cardiologists, the proposed instrument has some limitations and need further clinical examinations. An improvement is needed in terms of a standard procedure of image capture, analyze and classify the ECG signal automatically in an embedded system or mobile phone application. Another improvement for future works is clinical trials of the proposed instrument and comparing the trials with the gold standard examinations.

## Figures and Tables

**Table 1:** CNN architecture on classification part.

Layer (Type)	Output Shape	Parameter #
Flatten	(None, 25,088)	0
Fully Connected Layer	(None, 256)	6,422,784
LeakyReLU	(None, 256)	0
Dropout Layer (*p* = 0.5)	(None, 256)	0
Fully Connected Layer	(None, 256)	65,792
LeakyReLU	(None, 256)	0
Dropout Layer (*p* = 0.3)	(None, 256)	0
Fully Connected Layer	(None, 4)	1028
Total parameters: 6,489,604		
Trainable parameters: 6,489,604		
Non-trainable parameters: 0		

**Table 2:** CNN training hyperparameters.

Training Parameters	Description/Values
Optimizer	‘Adam’
Loss	cross entropy
Mini-Batch Size	16
Epochs	100
Training dataset	80%
Validation dataset	20%

**Table 3:** Comparison of accuracy and loss with successive epochs.

Class	Precision	Recall	F1-Score	Accuracy	Loss
Arrhythmia	0.99	0.95	0.97	0.9596	0.0859
Normal sinus	0.99	0.93	0.95		
Sudden death	1.00	1.00	1.00		
Supraventricular arrhythmia	0.91	0.96	0.93		

## Supplementary Materials

The video of the proposed real-time ECG image prediction can be downloaded at: https://www.youtube.com/watch?v=OkBuNrdZdgo (accessed on 31 January 2022).
